# A qualitative study of rural healthcare providers’ views of social, cultural, and programmatic barriers to healthcare access

**DOI:** 10.1186/s12913-022-07829-2

**Published:** 2022-04-02

**Authors:** Nicholas C. Coombs, Duncan G. Campbell, James Caringi

**Affiliations:** 1 School of Public & Community Health Sciences, University of Montana, 32 Campus Dr, Missoula, MT 59812 USA; 2 Department of Psychology, University of Montana, 32 Campus Dr, Missoula, MT 59812 USA

**Keywords:** Access to healthcare, Rural health, Qualitative methods

## Abstract

**Background:**

Ensuring access to healthcare is a complex, multi-dimensional health challenge. Since the inception of the coronavirus pandemic, this challenge is more pressing. Some dimensions of access are difficult to quantify, namely characteristics that influence healthcare services to be both acceptable and appropriate. These link to a patient’s acceptance of services that they are to receive and ensuring appropriate fit between services and a patient’s specific healthcare needs. These dimensions of access are particularly evident in rural health systems where additional structural barriers make accessing healthcare more difficult. Thus, it is important to examine healthcare access barriers in rural-specific areas to understand their origin and implications for resolution.

**Methods:**

We used qualitative methods and a convenience sample of healthcare providers who currently practice in the rural US state of Montana. Our sample included 12 healthcare providers from diverse training backgrounds and specialties. All were decision-makers in the development or revision of patients’ treatment plans. Semi-structured interviews and content analysis were used to explore barriers–appropriateness and acceptability–to healthcare access in their patient populations. Our analysis was both deductive and inductive and focused on three analytic domains: cultural considerations, patient-provider communication, and provider-provider communication. Member checks ensured credibility and trustworthiness of our findings.

**Results:**

Five key themes emerged from analysis: 1) a friction exists between aspects of patients’ rural identities and healthcare systems; 2) facilitating access to healthcare requires application of and respect for cultural differences; 3) communication between healthcare providers is systematically fragmented; 4) time and resource constraints disproportionately harm rural health systems; and 5) profits are prioritized over addressing barriers to healthcare access in the US.

**Conclusions:**

Inadequate access to healthcare is an issue in the US, particularly in rural areas. Rural healthcare consumers compose a hard-to-reach patient population. Too few providers exist to meet population health needs, and fragmented communication impairs rural health systems’ ability to function. These issues exacerbate the difficulty of ensuring acceptable and appropriate delivery of healthcare services, which compound all other barriers to healthcare access for rural residents. Each dimension of access must be monitored to improve patient experiences and outcomes for rural Americans.

## Background

Unequal access to healthcare services is an important element of health disparities in the United States [1], and there remains much about access that is not fully understood. The lack of understanding is attributable, in part, to the lack of uniformity in how access is defined and evaluated, and the extent to which access is often oversimplified in research [2]. Subsequently, attempts to address population-level barriers to healthcare access are insufficient, and access remains an unresolved, complex health challenge [3–5]. This paper presents a study that aims to explore some of the less well studied barriers to healthcare access, particularly those that influence healthcare acceptability and appropriateness.

In truth, healthcare access entails a complicated calculus that combines characteristics of individuals, their households, and their social and physical environments with characteristics of healthcare delivery systems, organizations, and healthcare providers. For one to fully ‘access’ healthcare, they must have the means to identify their healthcare needs and have available to them care providers and the facilities where they work. Further, patients must then reach, obtain, and use the healthcare services in order to have their healthcare needs fulfilled. Levesque and colleagues critically examined access conceptualizations in 2013 and synthesized all ways in which access to healthcare was previously characterized; Levesque et al. proposed five dimensions of access: approachability, acceptability, availability, affordability and appropriateness [2]. These refer to the ability to perceive, seek, reach, pay for, and engage in services, respectively.

According to Levesque et al.’s framework, the five dimensions combine to facilitate access to care or serve as barriers. Approachability indicates that people facing health needs understand that healthcare services exist and might be helpful. Acceptability represents whether patients see healthcare services as consistent or inconsistent with their own social and cultural values and worldviews. Availability indicates that healthcare services are reached both physically and in a timely manner. Affordability simplifies one’s capacity to pay for healthcare services without compromising basic necessities, and finally, appropriateness represents the fit between healthcare services and a patient’s specific healthcare needs [2]. This study focused on the acceptability and appropriateness dimensions of access.

Before the novel coronavirus (SARS-CoV-2; COVID-19) pandemic, approximately 13.3% of adults in the US did not have a usual source of healthcare [6]. Millions more did not utilize services regularly, and close to two-thirds reported that they would be debilitated by an unexpected medical bill [7–9]. Findings like these emphasized a fragility in the financial security of the American population [10]. These concerns were exacerbated by the pandemic when a sudden surge in unemployment increased un- and under-insurance rates [11]. Indeed, employer-sponsored insurance covers close to half of Americans’ total cost of illness [12]. Unemployment linked to COVID-19 cut off the lone outlet to healthcare access for many. Health-related financial concerns expanded beyond individuals, as healthcare organizations were unequipped to manage a simultaneous increase in demand for specialized healthcare services and a steep drop off for routine revenue-generating healthcare services [13]. These consequences of the COVID-19 pandemic all put additional, unexpected pressure on an already fragmented US healthcare system.

Other structural barriers to healthcare access exist in relation to the rural–urban divide. Less than 10% of US healthcare resources are located in rural areas where approximately 20% of the American population resides [14]. In a country with substantially fewer providers per capita compared to many other developed countries, persons in rural areas experience uniquely pressing healthcare provider shortages [15, 16]. Rural inhabitants also tend to have lower household income, higher rates of un- or under-insurance, and more difficulty with travel to healthcare clinics than urban dwellers [17]. Subsequently, persons in rural communities use healthcare services at lower rates, and potentially preventable hospitalizations are more prevalent [18]. This disparity often leads rural residents to use services primarily for more urgent needs and less so for routine care [19–21].

The differences in how rural and urban healthcare systems function warranted a federal initiative to focus exclusively on rural health priorities and serve as counterpart to Healthy People objectives [22]. The rural determinants of health, a more specific expression of general social determinants, add issues of geography and topography to the well-documented social, economic and political factors that influence all Americans’ access to healthcare [23]. As a result, access is consistently regarded as a top priority in rural areas, and many research efforts have explored the intersection between access and rurality, namely within its less understood dimensions (acceptability and appropriateness) [22].

### Acceptability-related barriers to care

Acceptability represents the dimension of healthcare access that affects a patient’s ability to seek healthcare, particularly linked to one’s professional values, norms and culture [2]. Access to health information is an influential factor for acceptable healthcare and is essential to promote and maintain a healthy population [24]. According to the Centers for Disease Control and Prevention, health literacy or a high ‘health IQ’ is the degree to which individuals have the ability to find, understand, and use information and services to inform health-related decisions and actions for themselves and others, which impacts healthcare use and system navigation [25]. The literature indicates that lower levels of health literacy contribute to health disparities among rural populations [26–28]. Evidence points to a need for effective health communication between healthcare organizations and patients to improve health literacy [24]. However, little research has been done in this area, particularly as it relates to technologically-based interventions to disseminate health information [29].

Stigma, an undesirable position of perceived diminished status in an individual’s social position, is another challenge that influences healthcare acceptability [30]. Those who may experience stigma fear negative social consequences in relation to care seeking. They are more likely to delay seeking care, especially among ethnic minority populations [31, 32]. Social media presents opportunities for the dissemination of misleading medical information; this runs further risk for stigma [33]. Stigma is difficult to undo, but research has shown that developing a positive relationship with a healthcare provider or organization can work to reduce stigma among patients, thus promoting healthcare acceptability [34].

A provider’s attempts to engage patients and empower them to be active decision-makers regarding their treatment has also been shown to improve healthcare acceptability. One study found that patients with heart disease who completed a daily diary of weight and self-assessment of symptoms, per correspondence with their provider, had better care outcomes than those who did not [35]. Engaging with household family members and involved community healers also mitigates barriers to care, emphasizing the importance of a team-based approach that extends beyond those who typically provide healthcare services [36, 37]. One study, for instance, explored how individuals closest to a pregnant woman affect the woman’s decision to seek maternity care; partners, female relatives, and community health-workers were among the most influential in promoting negative views, all of which reduced a woman’s likelihood to access care [38].

### Appropriateness-related barriers to care

Appropriateness marks the dimension of healthcare access that affects a patient’s ability to engage, and according to Levesque et al., is of relevance once all other dimensions (the ability to perceive, seek, reach and pay for) are achieved [2]. The ability to engage in healthcare is influenced by a patient’s level of empowerment, adherence to information, and support received by their healthcare provider. Thus, barriers to healthcare access that relate to appropriateness are often those that indicate a breakdown in communication between a patient with their healthcare provider. Such breakdown can involve a patient experiencing miscommunication, confrontation, and/or a discrepancy between their provider’s goals and their own goals for healthcare. Appropriateness represents a dimension of healthcare access that is widely acknowledged as an area in need of improvement, which indicates a need to rethink how healthcare providers and organizations can adapt to serve the healthcare needs of their communities [39]. This is especially true for rural, ethnic minority populations, which disproportionately experience an abundance of other barriers to healthcare access. Culturally appropriate care is especially important for members of minority populations [40–42]. Ultimately, patients value a patient-provider relationship characterized by a welcoming, non-judgmental atmosphere [43, 44]. In rural settings especially, level of trust and familiarity are common factors that affect service utilization [45]. Evidence suggests that kind treatment by a healthcare provider who promotes patient-centered care can have a greater overall effect on a patient’s experience than a provider’s degree of medical knowledge or use of modern equipment [46]. Of course, investing the time needed to nurture close and caring interpersonal connections is particularly difficult in under-resourced, time-pressured rural health systems [47, 48].

### Rationale

The most effective way to evaluate access to healthcare largely depends on which dimensions are explored. For instance, a population-based survey can be used to measure the barrier of healthcare affordability. Survey questions can inquire directly about health insurance coverage, care-related financial burden, concern about healthcare costs, and the feared financial impacts of illness and/or disability. Many national organizations have employed such surveys to measure affordability-related barriers to healthcare. For example, a question may ask explicitly about financial concerns: ‘If you get sick or have an accident, how worried are you that you will not be able to pay your medical bills?’ [49]. Approachability and availability dimensions of access are also studied using quantitative analysis of survey questions, such as ‘Is there a place that you usually go to when you are sick or need advice about your health?’ or ‘Have you ever delayed getting medical care because you couldn’t get through on the telephone?’ In contrast, the remaining two dimensions–acceptability and appropriateness–require a qualitative approach, as the social and cultural factors that determine a patient’s likelihood of accepting aspects of the services that are to be received (acceptability) and the fit between those services and the patient’s specific healthcare needs (appropriateness) can be more abstract [50, 51]. In social science, qualitative methods are appropriate to generate knowledge of what social events mean to individuals and how those individuals interact within them; these methods allow for an exploration of depth rather than breadth [52, 53]. Qualitative methods, therefore, are appropriate tools for understanding the depth of healthcare providers’ experiences in the inherently social context of seeking and engaging in healthcare.

In sum, acceptability- and appropriateness-related barriers to healthcare access are multi-layered, complex and abundant. Ensuring access becomes even more challenging if structural barriers to access are factored in. In this study, we aimed to explore barriers to healthcare access among persons in Montana, a historically underserved, under-resourced, rural region of the US. Montana is the fourth largest and third least densely populated state in the country; more than 80% of Montana counties are classified as non-core (the lowest level of urban/rural classification), and over 90% are designated as health professional shortage areas [54, 55]. Qualitative methods supported our inquiry to explore barriers to healthcare access related to acceptability and appropriateness.

## Methods

### Participants

Qualitative methods were utilized for this interpretive, exploratory study because knowledge regarding barriers to healthcare access within Montana’s rural health systems is limited. We chose Montana healthcare providers, rather than patients, as the population of interest so we may explore barriers to healthcare access from the perspective of those who serve many persons in rural settings. Inclusion criteria required study participants to provide direct healthcare to patients at least one-half of their time. We defined ‘provider’ as a healthcare organization employee with clinical decision-making power and the qualifications to develop or revise patients’ treatment plans. In an attempt to capture a group of providers with diverse experience, we included providers across several types and specialties. These included advanced practice registered nurses (APRNs), physicians (MDs and DOs), and physician assistants (PAs) who worked in critical care medicine, emergency medicine, family medicine, hospital medicine, internal medicine, pain medicine, palliative medicine, pediatrics, psychiatry, and urgent care medicine. We also included licensed clinical social workers (LCSWs) and clinical psychologists who specialize in behavioral healthcare provision.

### Recruitment and Data Collection

We recruited participants via email using a snowball sampling approach [56]. We opted for this approach because of its effectiveness in time-pressured contexts, such as the COVID-19 pandemic, which has made healthcare provider populations hard to reach [57]. Considering additional constraints with the pandemic and the rural nature of Montana, interviews were administered virtually via Zoom video or telephone conferencing with Zoom’s audio recording function enabled. All interviews were conducted by the first author between January and September 2021. The average length of interviews was 50 min, ranging from 35 to 70 min. There were occasional challenges experienced during interviews (poor cell phone reception from participants, dropped calls), in which case the interviewer remained on the line until adequate communication was resumed. All interviews were included for analysis and transcribed verbatim into NVivo Version 12 software. All qualitative data were saved and stored on a password-protected University of Montana server. Hard-copy field notes were securely stored in a locked office on the university’s main campus.

### Procedure

Data analysis included a deductive followed by an inductive approach. This dual analysis adheres to Levesque’s framework for qualitative methods, which is discussed in the Definition of Analytic Domains sub-section below. Original synthesis of the literature informed the development of our initial deductive codebook. The deductive approach was derived from a theory-driven hypothesis, which consisted of synthesizing previous research findings regarding acceptability- and appropriateness-related barriers to care. Although the locations, patient populations and specific type of healthcare services varied by study in the existing literature, several recurring barriers to healthcare access were identified. We then operationalized three analytic domains based on these findings: cultural considerations, patient-provider communication, and provider-provider communication. These domains were chosen for two reasons: 1) the terms ‘culture’ and ‘communication’ were the most frequently documented characteristics across the studies examined, and 2) they each align closely with the acceptability and appropriateness dimensions of access to healthcare, respectively. In addition, ‘culture’ is included in the definition of acceptability and ‘communication’ is a quintessential aspect of appropriateness. These domains guided the deductive portion of our analysis, which facilitated the development of an interview guide used for this study.

Interviews were semi-structured to allow broad interpretations from participants and expand the open-ended characterization of study findings. Data were analyzed through a flexible coding approach proposed by Deterding and Waters [58]. Qualitative content analysis was used, a method particularly beneficial for analyzing large amounts of qualitative data collected through interviews that offers possibility of quantifying categories to identify emerging themes [52, 59]. After fifty percent of data were analyzed, we used an inductive approach as a formative check and repeated until data saturation, or the point at which no new information was gathered in interviews [60]. At each point of inductive analysis, interview questions were added, removed, or revised in consideration of findings gathered [61]. The Standards for Reporting Qualitative Research (SRQR) was used for reporting all qualitative data for this study [62]. The first and third authors served as primary and secondary analysts of the qualitative data and collaborated to triangulate these findings. An audit approach was employed, which consisted of coding completed by the first author and then reviewed by the third author. After analyses were complete, member checks ensured credibility and trustworthiness of findings [63]. Member checks consisted of contacting each study participant to explain the study’s findings; one-third of participants responded and confirmed all findings. All study procedures were reviewed and approved by the Human Subjects Committee of the authors’ institution’s Institutional Review Board.

### Definitions of Analytic Domains

#### Cultural Considerations

Western health systems often fail to consider aspects of patients’ cultural perspectives and histories. This can manifest in the form of a providers’ lack of cultural humility. Cultural humility is a process of preventing imposition of one’s worldview and cultural beliefs on others and recognizing that everyone’s conception of the world is valid. Humility cultivates sensitive approaches in treating patients [64]. A lack of cultural humility impedes the delivery of acceptable and appropriate healthcare [65], which can involve low empathy or respect for patients, or dismissal of culture and traditions as superstitions that interfere with standard treatments [66, 67]. Ensuring cultural humility among all healthcare employees is a step toward optimal healthcare delivery. Cultural humility is often accomplished through training that can be tailored to particular cultural- or gender-specific populations [68, 69]. Since cultural identities and humility have been marked as factors that can heavily influence patients’ access to care, cultural considerations composed our first analytic domain. To assess this domain, we asked participants how they address the unique needs of their patients, how they react when they observe a cultural behavior or attitude from a patient that may not directly align with their treatment plan, and if they have received any multicultural training or training on cultural considerations in their current role.

### Patient-provider communication

Other barriers to healthcare access can be linked to ineffective patient-provider communication. Patients who do not feel involved in healthcare decisions are less likely to adhere to treatment recommendations [70]. Patients who experience communication difficulties with providers may feel coerced, which generates disempowerment and leads patients to employ more covert ways of engagement [71, 72]. Language barriers can further compromise communication and hinder outcomes or patient progress [73, 74]. Any miscommunication between a patient and provider can affect one’s access to healthcare, namely affecting appropriateness-related barriers. For these reasons, patient-provider communication composed our second analytic domain. We asked participants to highlight the challenges they experience when communicating with their patients, how those complications are addressed, and how communication strategies inform confidentiality in their practice. Confidentiality is a core ethical principle in healthcare, especially in rural areas that have smaller, interconnected patient populations [75].

### Provider-Provider Communication

A patient’s journey through the healthcare system necessitates sufficient correspondence between patients, primary, and secondary providers after discharge and care encounters [76]. Inter-provider and patient-provider communication are areas of healthcare that are acknowledged to have some gaps. Inconsistent mechanisms for follow up communication with patients in primary care have been documented and emphasized as a concern among those with chronic illness who require close monitoring [68, 77]. Similar inconsistencies exist between providers, which can lead to unclear care goals, extended hospital stays, and increased medical costs [78]. For these reasons, provider-provider communication composed our third analytic domain. We asked participants to describe the approaches they take to streamline communication after a patient’s hospital visit, the methods they use to ensure collaborative communication between primary or secondary providers, and where communication challenges exist.

## Results

### Healthcare provider characteristics

Our sample included 12 providers: four in family medicine (1 MD, 1 DO, 1 PA & 1 APRN), three in pediatrics (2 MD with specialty in hospital medicine & 1 DO), three in palliative medicine (2 MDs & 1 APRN with specialty in wound care), one in critical care medicine (DO with specialty in pediatric pulmonology) and one in behavioral health (1 LCSW with specialty in trauma). Our participants averaged 9 years (range 2–15) as a healthcare provider; most reported more than 5 years in their current professional role. The diversity of participants extended to their patient populations as well, with each participant reporting a unique distribution of age, race and level of medical complexity among their patients. Most participants reported that a portion of their patients travel up to five hours, sometimes across county- or state-lines, to receive care.

### Theme 1: A friction exists between aspects of patients’ rural identities and healthcare systems

Our participants comprised a collection of medical professions and reported variability among health-related reasons their patients seek care. However, most participants acknowledged similar characteristics that influence their patients’ challenges to healthcare access. These identified factors formed categories from which the first theme emerged. There exists a great deal of ‘rugged individualism’ among Montanans, which reflects a self-sufficient and self-reliant way of life. Stoicism marked a primary factor to characterize this quality. One participant explained:

*True Montanans are difficult to treat medically because they tend to be a tough group. They don’t see doctors. They don’t want to go, and they don’t want to be sick. That’s an aspect of Montana that makes health culture a little bit difficult.*

Another participant echoed this finding by stating:

*The backwoods Montana range guy who has an identity of being strong and independent probably doesn’t seek out a lot of medical care or take a lot of medications. Their sense of vitality, independence and identity really come from being able to take care and rely on themselves. When that is threatened, that’s going to create a unique experience of illness.*

Similar responses were shared by all twelve participants; stoicism seemed to be heavily embedded in many patient populations in Montana and serves as a key determinant of healthcare acceptability. There are additional factors, however, that may interact with stoicism but are multiply determined. Stigma is an example of this, presented in this context as one’s concern about judgement by the healthcare system. Respondents were openly critical of this perception of the healthcare system as it was widely discussed in interviews. One participant stated:

*There is a real perception of a punitive nature in the medical community, particularly if I observe a health issue other than the primary reason for one’s hospital visit, whether that may be predicated on medical neglect, delay of care, or something that may warrant a report to social services. For many of the patients and families I see, it’s not a positive experience and one that is sometimes an uphill barrier that I work hard to circumnavigate.*

Analysis of these factors suggest that low use of healthcare services may link to several characteristics, including access problems. Separately, a patient’s perceived stigma from healthcare providers may also impact a patient’s willingness to receive services. One participant put it best by stating

*Sometimes, families assume that I didn’t want to see them because they will come in for follow up to meet with me but end up meeting with another provider, which is frustrating because I want to maintain patients on my panel but available time and resource occasionally limits me from doing so. It could be really hard adapting to those needs on the fly, but it’s an honest miss.*

When a patient arrives for a healthcare visit and experiences this frustration, it may elicit a patient’s perceptions of neglect or disorganization. This ‘honest miss’ may, in turn, exacerbate other acceptable-related barriers to care.

### Theme 2: Facilitating access to healthcare requires application of and respect for cultural differences

The biomedical model is the standard of care utilized in Western medicine [79, 80]. However, the US comprises people with diverse social and cultural identities that may not directly align with Western conceptions of health and wellness. Approximately 11.5% of the Montana population falls within an ethnic minority group. 6.4% are of American Indian or Alaska Native origin, 0.5% are of Black or African American origin, 0.8% are of Asian origin and 3.8% are of multiple or other origins. [81]. Cultural insensitivity is acknowledged in health services research as an active deterrent for appropriate healthcare delivery [65]. Participants for this study were asked how they react when a patient brings up a cultural attitude or behavior that may impact the proposed treatment plan. Eight participants noted a necessity for humility when this occurs. One participant conceptualized this by stating:

*When this happens, I learn about individuals and a way of life that is different to the way I grew up. There is a lot of beauty and health in a non-patriarchal, non-dominating, non-sexist framework, and when we can engage in such, it is really expansive for my own learning process.*

The participants who expressed humility emphasized that it is best to work in tandem with their patient, congruently. Especially for those with contrasting worldviews, a provider and a patient working as a team poses an opportunity to develop trust. Without it, a patient can easily fall out of the system, further hindering their ability to access healthcare services in the future. One participant stated:

*The approach that ends up being successful for a lot of patients is when we understand their modalities, and they have a sense we understand those things. We have to show understanding and they have to trust. From there, we can make recommendations to help get them there, not decisions for them to obey, rather views based on our experiences and understanding of medicine.*

Curiosity was another reaction noted by a handful of participants. One participant said:

*I believe patients and their caregivers can be engaged and loving in different ways that don’t always follow the prescribed approach in the ways I’ve been trained, but that doesn’t necessarily mean that they are detrimental. I love what I do, and I love learning new things or new approaches, but I also love being surprised. My style of medicine is not to predict peoples’ lives, rather to empower and support what makes life meaningful for them.*

Participants mentioned several other characteristics that they use in practice to prevent cultural insensitivity and support a collaborative approach to healthcare. Table 1 lists these facilitating characteristics and quotes to explain the substance of their benefit.

**Table 1:** Facilitating factors to react to cultural attitude/behavior that does not align with treatment path

Facilitating Characteristics	Representative Quotation
Humility	*It’s about having humility and always working on listening* *There used to be this idea of cultural competence, but that’s moved out the window. We are now working to have cultural humility, to have humbleness in what we do not know*
Curiosity	*One of the reasons I’ve been successful and sustained in this area where a lot of other providers have a really short tenure is because I respond with a lot of curiosity. A lot of things that we do in medicine now are somewhat wise tale or passed along. There’s a real component of culture in healthcare. I see challenges of that in both traditional and non-traditional medicine and often learn a lot from people who have been in the community identified as medicine men or women. In the end, we all have the same goal, right?* *One trait that is nurtured in our field (social work) and among people who choose our field is empathetic curiosity*
Caution	*I am very attentive to cultural elements in healthcare. My entire career has been working around under-privileged, poverty-stricken or racially diverse patients, and I think that I realized the reality of medicine’s shortcomings when it comes to cultural insensitivity*
Empowered resistance	*Reactive anger or resistance doesn’t help anyone, but empowered resistance… those are two separate things. When I hit an edge with people, we talk about their anger and resistance and then we figure out where to go together*
Alignment	*On a pragmatic level, we recognize that when nurturing any relationship and developing trust, there first needs to be alignment* *One thing I regularly remind myself of is that it’s not about me. Any tension that I’m feeling is nothing compared to the distrust that the family is feeling. After the pressure is taken off, align, align, align, align*
Humor	*My goal is to help people become kinder, compassionate, and more open first to themselves, then to other people, so there’s a lot of humor when I reach an edge*

Consensus among participants indicated that the use of these protective factors to promote cultural sensitivity and apply them in practice is not standardized. When asked, all but two participants said they had not received any culturally-based training since beginning their practice. Instead, they referred to developing skills through “on the job training” or “off the cuff learning.” The general way of medicine, one participant remarked, was to “throw you to the fire.” This suggested that use of standardized cultural humility training modules for healthcare providers was not common practice. Many attributed this to time constraints.

Individual efforts to gain culturally appropriate skills or enhance cultural humility were mentioned, however. For example, three participants reported that they attended medical conferences to discuss cultural challenges within medicine, one participant sought out cultural education within their organization, and another was invited by Native American community members to engage in traditional peace ceremonies. Participants described these additional efforts as uncommon and outside the parameters of a provider’s job responsibilities, as they require time commitments without compensation.

Additionally, eight participants said they share their personal contact information with patients so they may call them directly for medical needs. The conditions and frequency with which this is done was variable and more common among providers in specialized areas of medicine or those who described having a manageable patient panel. All who reported that they shared their personal contact information described it as an aspect of rural health service delivery that is atypical in other, non-rural healthcare systems.

### Theme 3: Communication between healthcare providers is systematically fragmented

Healthcare is complex and multi-disciplinary, and patients’ treatment is rarely overseen by a single provider [82]. The array of provider types and specialties is vast, as is the range of responsibilities ascribed to providers. Thus, open communication among providers both within and between healthcare systems is vital for the success of collaborative healthcare [83]. Without effective communication achieved between healthcare providers, the appropriate delivery of healthcare services may be become compromised. Our participants noted that they face multiple challenges that complicate communication with other providers. Miscommunication between departments, often implicating the Emergency Department (ED), was a recurring point noted among participants. One participant who is a primary care physician said:

*If one of my patients goes to the ER, I don’t always get the notes. They’re supposed to send them to the patient’s primary care doc. The same thing happens with general admissions, but again, I often find out from somebody else that my patient was admitted to the hospital.*

This failure to communicate can negatively impact the patient, particularly if time sensitivity or medical complexity is essential to treatment. A patient’s primary care physician is the most accurate source of their medical history; without an effective way to obtain and synthesize a patient’s health information, there may be increased risk of medical error. One participant in a specialty field stated:

*One of the biggest barriers I see is obtaining a concise description of a patient’s history and needs. You can imagine if you’re a mom and you’ve got a complicated kid. You head to the ER. The ER doc looks at you with really wide eyes, not knowing how to get information about your child that’s really important.*

This concern was highlighted with a specific example from a different participant:

*I have been unable to troubleshoot instances when I send people to the ER with a pretty clear indication for admission, and then they’re sent home. For instance, I had an older fellow with pretty severe chronic kidney disease. He presented to another practitioner in my office with shortness of breath and swelling and appeared to have newly onset decompensated heart failure. When I figured this out, I sent him to the ER, called and gave my report. The patient later came back for follow up to find out not only that they had not been admitted but they lost no weight with outpatient* dialysis*. I feel like a real opportunity was missed to try to optimize the care of the patient simply because there was poor communication between myself and the ER. This poor guy… He ended up going to the ER four times before he got admitted for COVID-19.*

In some cases, communication breakdown was reported as the sole cause of a poor outcome. When communication is effective, each essential member of the healthcare team is engaged and collaborating with the same information. Some participants called this process ‘rounds’ when a regularly scheduled meeting is staged between a group of providers to ensure access to accurate patient information. Accurate communication may also help build trust and improve a patient’s experience. In contrast, ineffective communication can result in poor clarity regarding providers’ responsibilities or lost information. Appropriate delivery of healthcare considers the fit between providers and a patient’s specific healthcare needs; the factors noted here suggest that provider-provider miscommunication can adversely affect this dimension of healthcare access.

Another important mechanism of communication is the sharing of electronic medical records (EMRs), a process that continues to shift with technological advances. Innovation is still recent enough, however, for several of our study participants to be able to recall a time when paper charts were standard. Widespread adoption and embrace of the improvements inherent in electronic medical records expanded in the late 2000’s [84]. EMRs vastly improved the ability to retain, organize, safeguard, and transfer health information. Every participant highlighted EMRs at one point or another and often did so with an underlying sense of anger or frustration. Systematic issues and problems with EMRs were discussed. One participant provided historical context to such records:

*Years back, the government aimed to buy an electronic medical record system, whichever was the best, and a number of companies created their own. Each were a reasonable system, so they all got their checks and now we have four completely separate operating systems that do not talk to each other. The idea was to make a router or some type of relay that can share information back and forth. There was no money in that though, so of course, no one did anything about it. Depending on what hospital, clinic or agency you work for, you will most likely work within one of these systems. It was a great idea; it just didn’t get finished.*

Seven participants confirmed these points and their impacts on making coordination more difficult, relying on outdated communication strategies more often than not. Many noted this even occurs between facilities within the same city and in separate small metropolitan areas across the state. One participant said:

*If my hospital decides to contract with one EMR and the hospital across town contracts with another, correspondence between these hospitals goes back to traditional faxing. As a provider, you’re just taking a ‘fingered crossed’ approach hoping that the fax worked, is picked up, was put in the appropriate inbox and was actually looked at. Information acquisition and making sure it’s timely are unforeseen between EMRs.*

Participants reported an “astronomic” number of daily faxes and telephone calls to complete the communication EMRs were initially designed to handle. These challenges are even more burdensome if a patient moves from out of town or out of state; obtaining their medical records was repeatedly referred to as a “chore” so onerous that it often remains undone. Another recurring concern brought up by participants regarded accuracy within EMRs to lend a false sense of security. They are not frequently updated, not designed to be family-centered and not set up to do anything automatically. One participant highlighted these limitations by stating:

*I was very proud of a change I made in our EMR system [EPIC], even though it was one I never should have had to make. I was getting very upset because I would find out from my nursing assistant who read the obituary that one of my patients had died. There was a real problem with the way the EMR was notifying PCP’s, so I got an EPIC-level automated notification built into our EMR so that any time a patient died, their status would be changed to deceased and a notification would be sent to their PCP. It’s just really awful to find out a week later that your patient died, especially when you know these people and their families really well. It’s not good care to have blind follow up.*

Whether it be a physical or electronic miscommunication between healthcare providers, the appropriate delivery of healthcare can be called to question

### Theme 4: Time and resource constraints disproportionately harm rural health systems

Several measures of system capacity suggest the healthcare system in the US is under-resourced. There are fewer physicians and hospital beds per capita compared to most comparable countries, and the growth of healthcare provider populations has stagnated over time [15]. Rural areas, in particular, are subject to resource limitations [16]. All participants discussed provider shortages in detail. They described how shortages impact time allocation in their day-to-day operations. Tasks like patient intakes, critical assessments, and recovering information from EMRs take time, of which most participants claimed to not have enough of. There was also a consensus in having inadequate time to spend on medically complex cases. Time pressures were reported to subsequently influence quality of care. One participant stated:

*With the constant pace of medicine, time is not on your side. A provider cannot always participate in an enriching dialogue with their patients, so rather than listen and learn, we are often coerced into the mindset of ‘getting through’ this patient so we can move on. This echoes for patient education during discharge, making the whole process more arduous than it otherwise could be if time and resources were not as sparse.*

Depending on provider type, specialty, and the size of patient panels, four participants said they have the luxury of extending patient visits to 40 + minutes. Any flexibility with patient visits was regarded as just that: a luxury. Very few providers described the ability to coordinate their schedules as such. This led some study participants to limit the number of patients they serve. One participant said:

*We simply don’t have enough clinicians, which is a shame because these people are really skilled, exceptional, brilliant providers but are performing way below their capacity. Because of this, I have a smaller case load so I can engage in a level of care that I feel is in the best interest of my patients. Everything is a tradeoff. Time has to be sacrificed at one point or another. This compromise sets our system up to do ‘ok’ work, not great work.*

Of course, managing an overly large number of patients with high complexity is challenging. Especially while enduring the burden of a persisting global pandemic, participants reflected that the general outlook of administering healthcare in the US is to “do more with less.” This often forces providers to delegate responsibilities, which participants noted has potential downsides. One participant described how delegating patient care can cause problems.

*Very often will a patient schedule a follow up that needs to happen within a certain time frame, but I am unable to see them myself. So, they are then placed with one of my mid-level providers. However, if additional health issues are introduced, which often happens, there is a high-risk of bounce-back or need to return once again to the hospital. It’s an inefficient vetting process that falls to people who may not have specific training in the labs and imaging that are often included in follow up visits. Unfortunately, it’s a forlorn hope to have a primary care physician be able to attend all levels of a patient’s care.*

Several participants described how time constraints stretch all healthcare staff thin and complicate patient care. This was particularly important among participants who reported having a patient panel exceeding 1000. There were some participants, however, who praised the relationships they have with their nurse practitioners and physician’s assistants and mark transparency as the most effective way to coordinate care. Collectively, these clinical relationships were built over long standing periods of time, a disadvantage to providers at the start of their medical career. All but one participant with over a decade of clinical experience mentioned the usefulness of these relationships. The factors discussed in Theme 4 are directly linked to the Availability dimension of access to healthcare. A patient’s ability to reach care is subject to the capacity of their healthcare provider(s). Additionally, further analysis suggests these factors also link to the Appropriateness dimension because the quality of patient-provider relationships may be negatively impacted if a provider’s time is compromised.

### Theme 5: Profits are prioritized over addressing barriers to healthcare access in the US.

The US healthcare system functions partially for-profit in the public and private sectors. The federal government provides funding for national programs such as Medicare, but a majority of Americans access healthcare through private employer plans [85]. As a result, uninsurance rates influence healthcare access. Though the rate of the uninsured has dropped over the last decade through expansion of the Affordable Care Act, it remains above 8 percent [86]. Historically, there has been ethical criticism in the literature of a for-profit system as it is said to exacerbate healthcare disparities and constitute unfair competition against nonprofit institutions. Specifically, the US healthcare system treats healthcare as a commodity instead of a right, enables organizational controls that adversely affect patient-provider relationships, undermines medical education, and constitutes a medical-industrial complex that threatens influence on healthcare-related public policy [87]. Though unprompted by the interviewer, participants raised many of these concerns. One participant shared their views on how priorities stand in their practice:

*A lot of the higher-ups in the healthcare system* where *I work see each patient visit as a number. It’s not that they don’t have the capacity to think beyond that, but that’s what their role is, making sure we’re profitable. That’s part of why our healthcare system in the US is as broken as it is. It’s accentuated focus on financially and capitalistically driven factors versus understanding all these other barriers to care.*

Eight participants echoed a similar concept, that addressing barriers to healthcare access in their organizations is largely complicated because so much attention is directed on matters that have nothing to do with patients. A few other participants supported this by alluding to a “cherry-picking” process by which those at the top of the hierarchy devote their attention to the easiest tasks. One participant shared an experience where contrasting work demands between administrators and front-line clinical providers produces adverse effects:

*We had a new* administrator *in our hospital. I had been really frustrated with the lack of cultural awareness and curiosity from our other leaders in the past, so I offered to* meet and take them *on a tour of the reservation. This was meant to introduce* them *to kids, families and Tribal leaders who live in the area and their interface with healthcare.* They *declined, which I thought was disappointing and eye-opening.*

Analysis of these factors suggest that those who work directly with patients understand patient needs better than those who serve in management roles. This same participant went on to suggest an ulterior motive for a push towards telemedicine, as administrators primarily highlight the benefit of billing for virtual visits instead of the nature of the visits themselves.

## Discussion

This study explored barriers and facilitators to healthcare access from the perspective of rural healthcare providers in Montana. Our qualitative analysis uncovered five key themes: 1) a friction exists between aspects of patients’ rural identities and healthcare systems; 2) facilitating access to healthcare requires application of and respect for cultural differences; 3) communication between healthcare providers is systematically fragmented; 4) time and resource constraints disproportionately harm rural health systems; and 5) profits are prioritized over addressing barriers to healthcare access in the US. Themes 2 and 3 were directly supported by earlier qualitative studies that applied Levesque’s framework, specifically regarding healthcare providers’ poor interpersonal quality and lack of collaboration with other providers that are suspected to result from a lack of provider training [67, 70]. This ties back to the importance of cultural humility, which many previous culture-based trainings have referred to as cultural competence. Cultural competence is achieved through a plethora of trainings designed to expose providers to different cultures’ beliefs and values but induces risk of stereotyping and stigmatizing a patient’s views. Therefore, cultural humility is the preferred idea, by which providers reflect and gain open-ended appreciation for a patient’s culture [88].

### Implications for Practice

Perhaps the most substantial takeaway is how embedded rugged individualism is within rural patient populations and how difficult that makes the delivery of care in rural health systems. We heard from participants that stoicism and perceptions of stigma within the system contribute to this, but other resulting factors may be influential at the provider- and organizational-levels. Stoicism and perceived stigma both appear to arise, in part, from an understandable knowledge gap regarding the care system. For instance, healthcare providers understand the relations between primary and secondary care, but many patients may perceive both concepts as elements of a single healthcare system [89]. Any issue experienced by a patient when tasked to see both a primary and secondary provider may result in a patient becoming confused [90]. This may also overlap with our third theme, as a disjointed means of communication between healthcare providers can exacerbate patients’ negative experiences. One consideration to improve this is to incorporate telehealth programs into an existing referral framework to reduce unnecessary interfacility transfers; telehealth programs have proven effective in rural and remote settings [91].

In fact, telehealth has been rolled out in a variety of virtual platforms throughout its evolution, its innovation matched with continued technological advancement. Simply put, telehealth allows health service delivery from a distance; it allows knowledge and practice of clinical care to be in a different space than a patient. Because of this, a primary benefit of telehealth is its impact on improving patient-centered outcomes among those living in rural areas. For instance, text messaging technology improves early infant diagnosis, adherence to recommended diagnostic testing, and participant engagement in lifestyle change interventions [92–94]. More sophisticated interventions have found their way into smartphone-based technology, some of which are accessible even without an internet connection [95, 96]. Internet accessibility is important because a number of study participants noted internet connectivity as a barrier for patients who live in low resource communities. Videoconferencing is another function of telehealth that has delivered a variety of health services, including those which are mental health-specific [97], and mobile health clinics have been used in rural, hard-to-reach settings to show the delivery of quality healthcare is both feasible and acceptable [98–100]. While telehealth has potential to reduce a number of healthcare access barriers, it may not always address the most pressing healthcare needs [101]. However, telehealth does serve as a viable, cost-effective alternative for rural populations with limited physical access to specialized services [102]. With time and resource limitations acknowledged as a key theme in our study, an emphasis on expanding telehealth services is encouraged as it will likely have significant involvement on advancing healthcare in the future, especially as the COVID-19 pandemic persists [103].

### Implications for Policy

One could argue that most of the areas of fragmentation in the US healthcare system can be linked to the very philosophy on which it is based: an emphasis on profits as highest priority. Americans are, therefore, forced to navigate a health service system that does not work solely in their best interests. It is not surprising to observe lower rates of healthcare usage in rural areas, which may be a result from rural persons’ negative views of the US healthcare system or a perception that the system does not exist to support wellness. These perceptions may interact with ‘rugged individualism’ to squelch rural residents’ engagement in healthcare. Many of the providers we interviewed for this study appeared to understand this and strived to improve their patients’ experiences and outcomes. Though these efforts are admirable, they may not characterize all providers who serve in rural areas of the US. From a policy standpoint, it is important to recognize these expansive efforts from providers. If incentives were offered to encourage maximum efforts be made, it may lessen burden due to physician burnout and fatigue. Of course, there is no easy fix to the persisting limit of time and resources for providers, problems that require workforce expansion. Ultimately, though, the current structure of the US healthcare system is failing rural America and doing little to help the practice of rural healthcare providers.

### Implications for Future Research

It is important for future health systems research efforts to consider issues that arise from both individual- and system-level access barriers and where the two intersect. Oftentimes, challenges that appear linked to a patient or provider may actually stem from an overarching system failure. If failures are critically and properly addressed, we may refine our understanding of what we can do in our professional spaces to improve care as practitioners, workforce developers, researchers and advocates. This qualitative study was exploratory in nature. It represents a step forward in knowledge generation regarding challenges in access to healthcare for rural Americans. Although mental health did not come up by design in this study, future efforts exploring barriers to healthcare access in rural systems should focus on access to mental healthcare. In many rural areas, Montana included, rates of suicide, substance use and other mental health disorders are highly prevalent. These characteristics should be part of the overall discussion of access to healthcare in rural areas. Optimally, barriers to healthcare access should continue to be explored through qualitative and mixed study designs to honor its multi-dimensional stature.

### Strengths and Limitations

It is important to note first that this study interviewed healthcare providers instead of patients, which served as both a strength and limitation. Healthcare providers were able to draw on numerous patient-provider experiences, enabling an account of the aggregate which would have been impossible for a patient population. However, accounts of healthcare providers’ perceptions of barriers to healthcare access for their patients may differ from patients’ specific views. Future research should examine acceptability- and appropriateness-related barriers to healthcare access in patient populations. Second, study participants were recruited through convenience sampling methods, so results may be biased towards healthcare providers who are more invested in addressing barriers to healthcare access. Particularly, the providers interviewed for this study represented a subset who go beyond expectations of their job descriptions by engaging with their communities and spending additional uncompensated time with their patients. It is likely that a provider who exhibits these behavioral traits is more likely to participate in research aimed at addressing barriers to healthcare access. Third, the inability to conduct face-to-face interviews for our qualitative study may have posed an additional limitation. It is possible, for example, that in-person interviews might have resulted in increased rapport with study participants. Notwithstanding this possibility, the remote interview format was necessary to accommodate health risks to the ongoing COVID-19 pandemic. Ultimately, given our qualitative approach, results from our study cannot be generalizable to all rural providers’ views or other rural health systems. In addition, no causality can be inferred regarding the influence of aspects of rurality on access. The purpose of this exploratory qualitative study was to probe research questions for future efforts. We also acknowledge the authors’ roles in the research, also known as reflexivity. The first author was the only author who administered interviews and had no prior relationships with all but one study participant. Assumptions and pre-dispositions to interview content by the first author were regularly addressed throughout data analysis to maintain study integrity. This was achieved by conducting analysis by unique interview question, rather than by unique participant, and recoding the numerical order of participants for each question. Our commitment to rigorous qualitative methods was a strength for the study for multiple reasons. Conducting member checks with participants ensured trustworthiness of findings. Continuing data collection to data saturation ensured dependability of findings, which was achieved after 10 interviews and confirmed after 2 additional interviews. We further recognize the heterogeneity in our sample of participants, which helped generate variability in responses. To remain consistent with appropriate means of presenting results in qualitative research however, we shared minimal demographic information about our study participants to ensure confidentiality.

## Conclusion

The divide between urban and rural health stretches beyond a disproportionate allocation of resources. Rural health systems serve a more complicated and hard-to-reach patient population. They lack sufficient numbers of providers to meet population health needs. These disparities impact collaboration between patients and providers as well as the delivery of acceptable and appropriate healthcare. The marker of rurality complicates the already cumbersome challenge of administering acceptable and appropriate healthcare and impediments stemming from rurality require continued monitoring to improve patient experiences and outcomes. Our qualitative study explored rural healthcare providers’ views on some of the social, cultural, and programmatic factors that influence access to healthcare among their patient populations. We identified five key themes: 1) a friction exists between aspects of patients’ rural identities and healthcare systems; 2) facilitating access to healthcare requires application of and respect for cultural differences; 3) communication between healthcare providers is systematically fragmented; 4) time and resource constraints disproportionately harm rural health systems; and 5) profits are prioritized over addressing barriers to healthcare access in the US. This study provides implications that may shift the landscape of a healthcare provider’s approach to delivering healthcare. Further exploration is required to understand the effects these characteristics have on measurable patient-centered outcomes in rural areas.

## Data Availability

The datasets generated and/or analyzed during the current study are not publicly available due to individual privacy could be compromised but are available from the corresponding author on reasonable request. Ethics approval and consent to participate. All study procedures and methods were carried out in accordance with relevant guidelines and regulations from the World Medical Association Declaration of Helsinki. Ethics approval was given by exempt review from the Institutional Review Board (IRB) at the University of Montana (IRB Protocol No.: 186–20). Participants received oral and written information about the study prior to interview, which allowed them to provide informed consent for the interviews to be recorded and used for qualitative research purposes. No ethical concerns were experienced in this study pertaining to human subjects. Consent for publication. The participants consented to the publication of de-identified material from the interviews.
